# Relationship between food insecurity and housing instability on quality of care and quality of life in adults with diabetes

**DOI:** 10.1371/journal.pone.0278650

**Published:** 2022-12-06

**Authors:** Elise Mosley-Johnson, Rebekah J. Walker, Sneha Nagavally, Laura Hawks, Sanjay Bhandari, Hannah Trasser, Jennifer A. Campbell, Leonard E. Egede

**Affiliations:** 1 Division of General Internal Medicine, Department of Medicine, Medical College of Wisconsin, Milwaukee, WI, United States of America; 2 Center for Advancing Population Science, Medical College of Wisconsin, Milwaukee, WI, United States of America; UNITED KINGDOM

## Abstract

**Objective:**

Examine the relationship between food and housing insecurities, quality of care and quality of life in adults with diabetes using a nationally representative data source.

**Methods:**

Data from 39,604 adults with diabetes who indicated if they experienced food and/or housing insecurity in the Behavioral Risk Factor Surveillance System (2014, 2015, 2017) was analyzed. Outcomes included quality of care (HbA1c test, eye exam, diabetes education, foot check) and quality of life (general health status, poor physical and mental health days, poor overall health days). Logistic models were run for each quality of care measure and linear models were run for each quality of life measure adjusting for socio-demographics, insurance status, and comorbidity count.

**Results:**

35.6% of adults with diabetes reported housing insecurity, 31.8% reported food insecurity, and 23.4% reported both. After adjustment, food and housing insecurity was significantly associated with lower odds of having an eye exam (housing:0.73, 95%CI:0.63,0.85; food:0.78, 95%CI:0.67,0.92; both:0.69, 95%CI:0.59,0.82), worse general health status (housing:-0.06 95%CI:-0.11,-0.01; food:-0.16, 95%CI:-0.21,-0.10; both:-0.14, 95%CI:-0.20,-0.09), and an increased number of poor mental health days (housing:1.73, 95%CI:0.83,2.63; food:2.08, 95%CI:1.16,3.00; both:1.97, 95%CI:1.05,2.90). Food insecurity was also associated with lower odds of receiving diabetes education (0.86, 95%CI:0.74,0.99) and an increased number of poor physical health days (0.95, 95%CI:0.14,1.76).

**Conclusion:**

Changes to our healthcare delivery system are critical to improving standards of care and quality of life in all populations and may require a shift towards consideration of overlapping social risk factors rather than the siloed approach currently used.

## Introduction

Diabetes, both diagnosed and undiagnosed, impacts more than 34 million Americans, or 13% of the adult population and is still a leading cause of morbidity and mortality in the United States as of 2020 [1]. Poorly controlled diabetes results in significant complications and is the primary cause of blindness, end stage kidney disease, and non-traumatic amputations among adults in the U.S. [1, 2]. Long-term complications of diabetes are also associated with lower quality of life [2]. Such complications can be prevented if recommended diabetes treatment goals for A1C, blood pressure, and LDL cholesterol levels are persistently met, however, studies suggest there has been no significant improvement in meeting diabetes treatment goals over time [3–5]. In addition, gaps in diabetes care are especially persistent for nonwhite adults with diabetes, with only 14% of Non-Hispanic Blacks and 18% of Hispanics achieving the recommended treatment goals compared to 25% of Non-Hispanic Whites [4, 5].

Evidence shows that social determinants of health, including unmet material needs such as food insecurity and housing instability, may be important drivers of diabetes outcomes [3, 6]. They have been associated with poor health outcomes in individuals with diabetes and may worsen disparities in diabetes outcomes [7–11]. Prior work found food insecurity is significantly associated with poor glycemic control, postponing needed medical care, poor medication adherence, increased emergency department use and hospitalizations, and lower self-efficacy in an individual’s belief in their ability to achieve diabetes treatment goals, especially for low-income persons with diabetes [12–19]. Similarly, for patients with diabetes, housing instability is associated with postponing needed medical care, poor medication adherence, lower self-efficacy, increases in emergency department use and hospitalizations, and not having a usual source of care [12, 13, 20].

Food and housing insecurity are not mutually exclusive experiences, with studies highlighting their relationship to each other, and reflecting the need to consider the combination of both insecurities rather than acting on isolated factors [21–23]. For example, a recent study on low-income Latino adults found that those reporting material need insecurities, including housing and food insecurity, were more likely to sacrifice their health for material needs and the tradeoffs made were associated with medication nonadherence due to cost, poor glycemic control, and barriers to access [24]. Similarly, another recent study examining social needs among patients with diabetes found a dose response relationship of three prevalent unmet needs (housing instability/quality, food insecurity, and healthcare transportation) on diabetes control, where patients with more unmet social needs were at highest risk for uncontrolled diabetes [25]. Studies examining patients with diabetes found associations between food insecurity and missed eye exams and diabetes-related vision loss [26]. However, little is known about how food insecurity affects other diabetes quality of care measures. Though housing instability may undermine diabetes control by disrupting routine diabetes self-care behaviors, as well by preventing patients from prioritizing self-care, investigation has focused primarily on access to care instead of quality of care [27, 28]. Both food and housing insecurity may additionally reduce quality of life for patients with diabetes. Food insecure adults with diabetes are more likely to report fair or poor overall health, lower satisfaction with life and have increased rates of depression, and diabetes distress [16–19]. In addition, housing insecure individuals are more likely to report their general health status as fair or poor [27, 28], but little work has been done on the quality of life for individuals with diabetes facing housing instability or adults with diabetes who are experiencing both food and housing insecurities.

To address these gaps in knowledge, we examined the relationship between food and housing insecurities and diabetes quality of care and quality of life using the Behavioral Risk Factor Surveillance System (BRFSS), a nationally representative survey of US adults. We hypothesized that individuals with food or housing insecurities and diabetes would be less likely to have participated in standard quality of care measures and would have poorer quality of life when compared to those who were food and housing secure.

## Materials and methods

### Data and sample

We analyzed participant responses collected from Behavioral Risk Factor Surveillance System (BRFSS) for years 2014 and 2015, the last years to include the optional social context module, and from the 2017 BRFSS which included an optional social determinants of health module. BRFSS is a state-based surveillance system that collects information about U.S. residents on their health behaviors, chronic conditions, and use of preventive services [29–31]. Additionally, BRFSS has incorporated optional questionnaire modules focusing on social determinants, including food and housing insecurities. For the purposes of our present analysis, we extracted data on housing insecurity and food insecurity from the optional social context (2014–2015) and social determinants of health (2017) modules of the questionnaire and matched this to core questionnaire information for the respective year [29, 30].

The survey question ‘Have you ever been told by a doctor you have diabetes?’ was used to identify participants with diabetes mellitus (n = 39,604). If respondent selected ‘pre-diabetic’, ‘borderline diabetes’ or ‘was diabetic only during pregnancy’ then they were not considered to have diabetes. This sample of adults with self-reported diabetes was used for the analysis and weighted using survey weights to be representative of the US population (N = 17,456,814).

### Outcome measures

#### Quality of care

- *HbA1c completed in the past 12 months*. Identified respondents who answered either ‘Yes’ or ‘No’ for survey question ‘If a health professional checked HbA1c at least once in the past 12 months.’
- *Dilated eye exam in the past 12 months*. Identified respondents who had dilated eye exam by provider if individuals reported at least one dilated eye exam in the past 12 months with response type ‘Yes’ vs ‘No’.
- *Diabetes education*. Identified respondents following who answered either ‘Yes’ or ‘No’ for survey question ‘Have you ever taken a course or class in how to manage your diabetes yourself?’
- *Foot check per day*. Identified respondents using survey question ‘About how often do you check your feet for any sores or irritations?’ Response type grouped into ‘Yes’ if examined any number of times per day or ‘No’ if not examined daily or never examined.

#### Quality of life

- *General health status*. Respondents answered the question ‘Would you say that in general your health is excellent, very good, good, fair, or poor?’ Response options were treated as a continuous variable with responses ranging from 1 (poor) to 5 (excellent) and thus higher numbers indicated better general health status.
- *Poor Physical Health Days*. Determined using survey item ‘Now thinking about your physical health, which includes physical illness and injury, for how many days during the past 30 days was your physical health not good?’ The variable was treated as continuous with responses ranging from 10 to 30 days.
- *Poor Mental Health Days*. Determined using survey item ‘Now thinking about your mental health, which includes stress, depression, and problems with emotions, for how many days during the past 30 days was your mental health not good?’ Variable was treated as continuous with responses ranging from 10 to 30 days.
- *Poor Overall Health Days*. Determined using survey item ‘During the past 30 days, for about how many days did poor physical or mental health keep your from doing your usual activities, such as self-care, work, or recreation?’ Variable was treated as continuous with responses ranging from 10 to 30 days.

### Independent variables

#### Housing insecurity

Assessed using the 2014–2015 survey question ‘How often in the past 12 months would you say you were worried or stressed about having enough money to pay your rent or mortgage?’ Response types ‘Rarely’, ‘Sometimes’, ‘Usually’ or ‘Always’ were categorized as ‘Yes’ and ‘Never’ categorized as ‘No’. For 2017 we used the survey question ‘During the last 12 months, was there a time when you were not able to pay your mortgage, rent or utility bills?’ with response type ‘Yes’ or ‘No’.

### Food insecurity

Assessed using the 2014–2015 survey question ‘How often in the past 12 months would you say you were worried or stressed about having enough money to buy nutritious meals? Response types ‘Rarely’, ‘Sometimes’, ‘Usually’ or ‘Always’ were categorized as ‘Yes’ and ‘Never’ was categorized as ‘No’. For 2017 we used survey question ‘In the last 12 months how often the food that I bought just didn’t last, and I didn’t have money to get more?’ Response type ‘Often true’, and ‘Sometimes true’ were categorized as ‘Yes’ while ‘Never true’ was categorized as ‘No’. The food security question used in the BRFSS is a simplified version of the USDA’s Current Population Survey food security supplement (CPS-FSS) and these two measures of food insecurity have been found to be highly correlated [32].

### Both housing and food insecurity

In order to capture the population that experienced both food insecurity and housing insecurity, we created a new variable which considers having both if, responses to both food insecurity and housing insecurity are ‘Yes’, else recorded as ‘No’.

### Covariates

Sociodemographic variables included age, sex (female vs male), race/ ethnicity (Non-Hispanic White, Non-Hispanic Black, Hispanic/other), level of education (less than high school education, high school graduate/GED, some college degree and college graduate), marital status (defined as married or not married), household income (less than $25,000, $25,000-$75,000 or more than $75,000), employment status (employed vs. unemployed), and insurance status (insured vs. uninsured). Comorbidities included in the survey (heart attack, angina, stroke, skin cancer, any other cancer, asthma, COPD, arthritis, depression, and kidney disease) were counted for each individual and included as a covariate for comorbidity burden. Covariates and comorbidities were selected based on prior literature listing potential confounders of the relationship between the variables.

### Statistical analysis

Descriptive statistics were used to calculate means ± standard errors and frequencies for continuous and categorical variables respectively to understand sociodemographic characteristics for the overall sample and by housing insecurity and food insecurity status. A series of regression models were run to investigate the association of both housing insecurity and food insecurity with each of the quality of care outcome measures (HbA1c test, eye exam, diabetes education and foot check) and quality of life outcome measures (general health status, poor physical health days, poor mental health days, and poor overall health days). First, logistic models were run for each quality of care outcome measure and housing insecurity, followed by each quality of care outcome measure and food insecurity, and finally each quality of care outcome measure and both food and housing insecurity. Second, linear models were run for each quality of life measure and housing insecurity, followed by each quality of life outcome measure and food insecurity, and finally each quality of life outcome measure and both food and housing insecurity. All models were run first unadjusted and then adjusted for age, sex, race/ethnicity, level of education, marital status, household income, employment status, insurance status, and comorbidity count. Complete statistical analysis was weighted to account for complex survey design using ‘survey’ package in R version-4.0.0 and statistical significance was assessed at p < 0.05.

## Results

Table 1 displays the sample demographics for adults with diabetes who completed the BRFSS survey overall and stratified by housing insecurity and food insecurity status. Overall, 35.6% of adults with diabetes reported housing insecurity, 31.8% reported food insecurity, and 23.4% reported both food and housing insecurity. The mean age was 60.82 years (standard deviation of 13.26), and the majority of respondents had a household income of $75,000 or less (81.4%) and were insured (92.5%). Over half the respondents were non-Hispanic White (68.9%), were unemployed (68.3%), and over one third graduated from high school or got their GED (33.5%). The mean comorbidity count was 1.84 (standard deviation of 1.64). For those reporting housing insecurity, over half had a household income of less than $25,000 or less (54.34%) and were unemployed (66.95%). For those reporting food insecurity, over half had a household income of less than $25,000 or less (63.60%) and were unemployed (72.53%).

**Table 1:** Sample demographics for adults with diabetes, BRFSS (n = 39,604).

	Overall (n = 39,604)	Housing Insecurity (n = 27,591)		Food Insecurity (n = 28,611)	
		No (n = 18,709)	Yes (n = 8,882)	No (n = 20,323)	Yes (n = 8,288)
Age, mean (s.d)	60.82 (13.26)	63.41 (12.51)	57.33 (12.69)	63.31 (12.43)	56.57 (13.15)
Sex, n (%)					
Female	22,225 (50.6%)	9,835 (48.20%)	5,654 (56.08%)	10,533 (46.72%)	5,612 (60.38%)
Male	17,364 (49.4%)	8,866 (51.80%)	3,227 (43.92%)	9,781 (53.28%)	2,676 (39.62%)
Race, n (%)					
Non-Hispanic White	29,398 (68.9%)	14,894 (73.23%)	5,984 (62.54%)	16,083 (73.05%)	5,500 (61.75%)
Non-Hispanic Black	6,196 (19.9%)	2,246 (15.97%)	1,952 (27.01%)	2,639 (17.28%)	1,795 (25.98%)
Hispanic/Other	3,296 (11.3%)	1,293 (10.80%)	802 (10.44%)	1,313 (9.67%)	839 (12.27%)
Education, n (%)					
Less than High School	4,789 (19.6%)	1,884 (16.74%)	1,423 (24.26%)	1,959 (16.20%)	1,539 (27.18%)
High School Grad/GED	13,433 (33.5%)	5,987 (32.78%)	3,125 (33.28%)	6,368 (32.42%)	3,112 (34.17%)
Some College	11,205 (30.0%)	5,354 (30.31%)	2,646 (30.69%)	5,887 (30.82%)	2,383 (29.29%)
College Graduate	10,037 (16.9%)	5,444 (20.17%)	1,669 (11.76%)	6,063 (20.57%)	1,232 (9.37%)
Marital Status, n (%)					
Not Married	20,263 (47.5%)	8,818 (43.11%)	5,111 (52.19%)	9,343 (41.75%)	5,292 (58.64%)
Household income, n (%)					
Less than $25,000	12,905 (40.9%)	4,943 (31.45%)	4,209 (54.34%)	4,986 (29.74%)	4,623 (63.60%)
$25,000-$75,000	13,399 (40.5%)	7,048 (44.26%)	2,822 (36.63%)	7,988 (46.07%)	2,075 (30.30%)
More than $75,000	5,798 (18.6%)	3,496 (24.30%)	646 (9.02%)	3,858 (24.19%)	331 (6.10%)
Employment Status, n (%)					
Unemployed	28,685 (68.3%)	13,590 (68.74%)	6,315 (66.95%)	14,418 (66.66%)	6,341 (72.53%)
Insurance Status, n (%)					
Not Insured	1,965 (7.5%)	673 (5.06%)	699 (11.36%)	734 (5.55%)	719 (11.75%)
Comorbidity Count, mean (s.d)	1.84 (1.64)	1.68 (1.54)	2.05 (1.70)	1.64 (1.52)	2.21 (1.72)

Table 2 displays the unadjusted and adjusted logistic regression models for the relationship between housing and food insecurity with quality of care in adults with diabetes. After adjustment, individuals who reported housing insecurity had a lower odds of having an eye exam (0.73, 95% CI, 0.63, 0.85). Those reporting food insecurity also had lower likelihood of having an eye exam (0.78, 95% CI, 0.67,0.92) in addition to a lower likelihood of receiving diabetes education (0.86, 95% CI, 0.74, 0.99). Individuals reporting both housing and food insecurity together also had lower odds of having an eye exam (0.69, 95% CI, 0.59, 0.82).

**Table 2:** Adjusted association between housing insecurity and food insecurity with quality of care measures in adults with diabetes, BRFSS (housing insecurity n = 8, 882; food insecurity n = 8,288).

	Unadjusted OR (95% Cl)	*p*-value	Adjusted OR (95% Cl)	*p*-value
**Housing Insecurity**	** **	** **	** **	** **
A1c Check	0.60*** (0.48, 0.74)	**<0.001**	0.81 (0.62, 1.05)	0.12
Eye Exam	0.58*** (0.51, 0.65)	**<0.001**	0.73*** (0.63, 0.85)	**<0.001**
Diabetes Education	0.98 (0.87, 1.10)	0.71	0.99 (0.87, 1.13)	0.35
Foot Check	1.14 (0.99, 1.31)	0.07	1.07 (0.92, 1.26)	0.39
**Food Insecurity**				
A1c Check	0.60*** (0.49, 0.74)	**<0.001**	0.92 (0.69, 1.23)	0.6
Eye Exam	0.59*** (0.52, 0.67)	**<0.001**	0.78** (0.67, 0.92)	**0.003**
Diabetes Education	0.83** (0.74, 0.94)	**0.003**	0.86* (0.74, 0.99)	**0.04**
Foot Check	**1.09 (0.95, 1.26)**	0.23	0.97 (0.82, 1.15)	0.74
**Both Housing & Food Insecurity**				
A1c Check	0.60*** (0.48, 0.75)	**<0.001**	0.81 (0.61, 1.07)	0.14
Eye Exam	0.55*** (0.48, 0.63)	**<0.001**	0.69*** (0.59, 0.82)	**<0.001**
Diabetes Education	0.86* (0.75, 0.98)	**0.03**	0.86 (0.74, 1.01)	0.06
Foot Check	**1.13 (0.96, 1.31)**	0.14	1.06 (0.89, 1.28)	0.5

Each model is adjusted for demographics, socioeconomic status, insurance status and comorbidity count

Table 3 displays the unadjusted and adjusted association between housing and food insecurity with quality of life in adults with diabetes. Prior to adjustment, housing insecurity, food insecurity, and both housing and food insecurity were significantly associated with all outcomes. After adjustment, housing insecurity was significantly associated with worse general health status (-0.06 95% CI, -0.11, -0.01) and higher number of poor mental health days (1.73, 95% CI, 0.83, 2.63). After adjustment, food insecurity was significantly associated with worse general health status (-0.16, 95% CI, -0.21, -0.10), higher number of poor physical health days (0.95, 95% CI, 0.14, 1.76), and a higher number of poor mental health days (2.08, 95% CI, 1.16, 3.00). After adjustment, both housing and food insecurity together were significantly associated with worse general health status (-0.14, 95% CI, -0.20, -0.09) and a higher number of poor mental health days (1.97, 95% CI, 1.05, 2.90).

**Table 3:** Adjusted association between housing insecurity and food insecurity with quality of life in adults with diabetes BRFSS (housing insecurity n = 8, 882; food insecurity n = 8,288).

	Unadjusted β estimates (95% Cl)	*p*-value	Adjusted β estimates (95% Cl)	*p*-value
**Housing Insecurity**	** **	** **	** **	** **
General Health Status	-0.27*** (-0.31, -0.22)	**<0.001**	-0.06* (-0.11, -0.01)	**0.02**
Poor Physical Health (days)	1.79*** (1.09, 2.48)	**<0.001**	0.64 (-0.14, 1.43)	0.11
Poor Mental Health (days)	2.94*** (2.13, 3.74)	**<0.001**	1.73*** (0.83, 2.63)	**<0.001**
Poor Overall Health (days)	1.53*** (0.68, 2.37)	**<0.001**	0.64 (-0.32, 1.60)	0.20
**Food Insecurity**			** **	** **
General Health Status	-0.45*** (-0.49, -0.40)	**<0.001**	-0.16*** (-0.21, -0.10)	**<0.001**
Poor Physical Health (days)	2.71*** (2.03, 3.39)	**<0.001**	0.95* (0.14, 1.76)	**0.03**
Poor Mental Health (days)	3.93*** (3.15, 4.71)	**<0.001**	2.08*** (1.16, 3.00)	**<0.001**
Poor Overall Health (days)	2.25*** (1.43, 3.06)	**<0.001**	0.73 (-0.25, 1.71)	0.15
**Both Housing & Food Insecurity**				
General Health Status	-0.39*** (-0.44, -0.34)	**<0.001**	-0.14*** (-0.20, -0.09)	**<0.001**
Poor Physical Health (days)	2.22*** (1.50, 2.94)	**<0.001**	0.82 (0.01, 1.63)	0.05
Poor Mental Health (days)	3.24*** (2.44, 4.05)	**<0.001**	1.97*** (1.05, 2.90)	**<0.001**
Poor Overall Health (days)	1.95*** (1.11, 2.79)	**<0.001**	0.92 (-0.03, 1.87)	0.06

Each model is adjusted for demographics, socioeconomic status, insurance status and comorbidity score

## Discussion

In this nationally representative sample of adults with diabetes, we found that housing and food insecurity were associated with lower odds of having an eye exam, worse general health status and increased number of poor physical and mental health days. Individuals facing both housing and food insecurity together were also less likely to receive an eye exam, more likely to report poor general health and report a higher number of poor mental health days. This study adds to the current literature by highlighting that while many of the diabetes quality of care measures were similar, specialized tests, such as eye exams, may be an important area of intervention for adults with food and housing insecurities. Eye exams are an important component in detecting early signs of diabetic retinopathy and preventing future vision loss in persons with diabetes [26]. Additionally, diabetes education is foundational to proper diabetes self-management and learning how to delay or avoid serious complications [33]. Future research should focus on how to increase access to more than primary care for diabetes management within the context of social risks being experienced.

In addition, this study highlights the need to consider the influence of food and housing insecurities on quality of life for adults with diabetes, and questions the need to consider these insecurities as isolated factors. While the impact of individual risk factors on health outcomes has been widely studied, there is also a growing recognition of the cumulative effect of having multiple social risk factors on health outcomes. For example, the higher rates of food insecurity among formerly homeless individuals living in permanent supportive housing far exceeded the general population [23]. This study found similar relationships across individual and combined food and housing insecurities, suggesting a more effective method for identifying risk in a clinical population may be the existence of any number of social risk factors. The current siloed approach to intervention development and delivery for either food or housing insecurities may fall short of having an impact on outcomes if they fail to address the cumulative impact of having unmet needs. A number of studies have investigated how to use multidisciplinary care teams, including peer navigators, social workers, and/or community health workers, to link adults with food insecurity to community supports and resources [34, 35]. Using food pantries, or other non-profit organizations that provide participants food assistance have also shown promising results in improving glycemic control, medication adherence, diet, physical activity, depression scores and diabetes distress [13, 36]. However, little research has focused on how to link individuals to resources or organizations that meet multiple social risks, which may be necessary for long-term impact.

Individual level interventions for patients with diabetes who face housing insecurity can be more difficult to implement, but work is ongoing. Incorporating Medical Legal Partnerships (MLPs) into federal healthcare programs have been recommended as one way to address unmet needs by providing patients with legal aid and assistance with finding social programs or government benefits [37]. Housing based interventions for diabetes have shown some promise, particularly in regard to diabetes prevention and health care utilization and access [38]. A study on supportive housing and diabetes outcomes among homeless adults in New York found that people who were placed in housing received improved diabetes care (HbA1c and lipid testing) and placement was associated with a reduction in new diabetes diagnoses when compared to unplaced homeless adults [39]. Several studies assessing Housing First programs in relationship to other health outcomes found participants reported significantly improved quality of life, spent less time homeless or hospitalized, and were more likely to utilize needed services for treatment, including medical, dental, and vision services [40, 41]. More work is needed to identify the cumulative effects of multiple social risk factors on outcomes and comprehensive and sustainable solutions that can address both the social and medical needs of adults with diabetes.

Policy level interventions that address health disparities by targeting social and non-medical care should be integrated with medical care based on the expanse of published literature linking social determinants of health to diabetes prevalence and outcomes. In a recent systematic review looking at nonmedical interventions for type 2 diabetes, researchers identified five policy opportunities backed by evidence that target social risk factors and health disparities: 1) the expansion of Medicaid to all states and the inclusion of coverage for food supplementation based on both medical and financial need; 2) ensuring health care access for vulnerable communities by preventing the closure of critical safety net hospitals and clinics due to financial pressures on health systems, such as seen during the COVID-19 pandemic; 3) legislation for new billing practices that protect uninsured patients, particularly those with diabetes that have high medical care needs and need protection from having to delay care due to cost or bankruptcy; 4) labor market policies targeting social mobility and social infrastructure, including increased minimum wage and paid time off; 5) expansion of subsidized housing, housing vouchers, and subsidized employment in areas with high unemployment rates [42].

### Limitations

Though this study used nationally representative data, there are several limitations to note. The first is that the data is cross-sectional and cannot be used to understand causality. Second measures were largely self-reported. While the self-reported diabetes measure used in this study has been shown to be a valid and reliable measure of diabetes, those who do not have a diagnosis or are unaware of their condition would not be included in the study sample [43]. Research on self-reported diabetes quality of care measures has specifically shown that eye examinations and A1c checks are likely to be overestimated when self-report data is used [44]. Future data should use datasets with both self-reported and biologic measures for diabetes to remove bias from quality of care measures and to include the population of individuals with undiagnosed diabetes. Lastly, the measure of both food and housing insecurities were assessed using only one question and may under-represent the range of experiences surrounding food and housing insecurities. However, research on BRFSS data has shown much support for the validity of its data and the BRFSS has historically been useful as an essential source of health information for states and local jurisdictions [45].

## Conclusions

Quality of care for individuals with diabetes in the U.S. in the past two decades has continued to fall short of diabetes care targets despite advances in care delivery models, technologies, and new diabetes medications [5]. In this study, food and housing insecurity was associated with fewer quality of care metrics, specifically eye exams and diabetes education, and with lower quality of life. Changes to our healthcare delivery system are critical to improving standards of care in all populations and may require a dramatic shift towards coordination with social and government services, and a consideration of multiple social risk factors rather than the siloed approach currently used. Interventions should be developed to be tailored to the unmet needs of the individual instead of being developed to address one unmet need over another.

## References

[1] Centers for Disease Control and Prevention. Atlanta, GA: Centers for Disease Control and Prevention, US Department of Health and Human Services. National Diabetes Statistics Report 2020: Estimates of Diabetes and Its Burden in the United States. Available from: https://www.cdc.gov/diabetes/pdfs/data/statistics/national-diabetes-statistics-report.pdf

[2] Centers for Disease Control and Prevention. National Diabetes Statistics Report: Estimates of Diabetes and Its Burden in the United States, 2014, Atlanta, GA: US Department of Health and Human Services; 2014. https://stacks.cdc.gov/view/cdc/23442/cdc_23442_DS1.pdf

[3] American Diabetes Association. 1. Improving Care and Promoting Health in Populations: Standards of Medical Care in Diabetes—2019. *Diabetes Care*. 2019;43(Suppl. 1):S37–S47 doi: 10.2337/dc19-S001 31862744 PMC11869376

[4] Ali MK, McKeever Bullard K, Saaddine JB et al. Achievement of goals in US diabetes care, 1999–2010. *N Engl J Med*. 2013; 368(17):1613–1624. doi: 10.1056/NEJMsa1213829 23614587

[5] Kazemian P, Shebl FM, McCann N, Walensky RP, Wexler DJ. Evaluation of the cascade of diabetes care in the United States, 2005–2016. *JAMA Intern Med*. 2019;179(10): 1376–1385. doi: 10.1001/jamainternmed.2019.2396 31403657 PMC6692836

[6] Walker RJ, Smalls BL, Campbell JA, Strom Williams J, Egede LE. Impact of social determinants of health on outcomes for type 2 diabetes: a systematic review. *Endocrine*. 2014. doi: 10.1007/s12020-014-0195-0 24532079 PMC7029167

[7] Trikkalinou A, Papazafiropoulou AK, Melidonis A. “Type 2 Diabetes and Quality of Life.” *World Journal of Diabetes* vol. 8,4 (2017): 120–129. doi: 10.4239/wjd.v8.i4.120 28465788 PMC5394731

[8] Gary-Webb TL, Baptiste-Roberts K, Pham L, Wesche-Thobaben J, Patricio J, Pi-Sunyer FX, et al. Neighborhood socioeconomic status, depression, and health status in the Look AHEAD (Action for Health in Diabetes) study. *BMC Public Health*. 2011 May 19;11:349. doi: 10.1186/1471-2458-11-349 ; PMCID: PMC3111582. 22182286 PMC3111582

[9] Abdurahman AA, Chaka EE, Nedjat S, Dorosty AR, Majdzadeh R. The association of household food insecurity with the risk of type 2 diabetes mellitus in adults: a systematic review and meta-analysis. Eur J Nutr. 2018. doi: 10.1007/s00394-018-1705-2 29721679

[10] Berkowitz SA, Karter AJ, Corbie-Smith G, et al. Food Insecurity, Food “Deserts,” and Glycemic Control in Patients With Diabetes: A Longitudinal Analysis. Diabetes Care. 2018;41(6):1188–1195. doi: 10.2337/dc17-1981 29555650 PMC5961388

[11] Berkowitz SA, Kalkhoran S, Edwards ST, Essien UR, Baggett TP. Unstable Housing and Diabetes-Related Emergency Department Visits and Hospitalization: A Nationally Representative Study of Safety-Net Clinic Patients. Diabetes Care. 2018;41(5):933–939. doi: 10.2337/dc17-1812 29301822 PMC5911783

[12] Myers CA. Understanding the importance of Food Insecurity among populations with diabetes. J Diabetes Complications. 2019;33(4):340–341. doi: 10.1016/j.jdiacomp.2018.12.008 30717894 PMC6615549

[13] Schroeder EB, Zeng C, Sterrett AT, Kimpo TK, Paolino AR, Steiner JF. The longitudinal relationship between food insecurity in older adults with diabetes and emergency department visits, hospitalizations, hemoglobin A1c, and medication adherence. J Diabetes Complications. 2019;33(4):289–295. doi: 10.1016/j.jdiacomp.2018.11.011 30717893 PMC6660013

[14] Kushel MB, Gupta R, Gee L, Haas JS. Housing instability and food insecurity as barriers to health care among low-income Americans. J Gen Intern Med. 2006;21(1):71–77. doi: 10.1111/j.1525-1497.2005.00278.x 16423128 PMC1484604

[15] Vijayaraghavan M, Jacobs EA, Seligman H, Fernandez A. The association between housing instability, food insecurity, and diabetes self-efficacy in low-income adults. J Health Care Poor Underserved. 2011;22(4):1279–1291. doi: 10.1353/hpu.2011.0131 22080709

[16] Gucciardi E, Vahabi M, Norris N, Del Monte JP, Farnum C. The Intersection between Food Insecurity and Diabetes: A Review. Curr Nutr Rep. 2014;3(4):324–332. doi: 10.1007/s13668-014-0104-4 25383254 PMC4218969

[17] Seligman HK, Jacobs EA, Lopez A, Tschann J, Fernandez A. Food insecurity and glycemic control among low-income patients with type 2 diabetes. Diabetes Care. 2012;35(2):233–238. doi: 10.2337/dc11-1627 22210570 PMC3263865

[18] Silverman J, Krieger J, Kiefer M, Hebert P, Robinson J, Nelson K. The Relationship Between Food Insecurity and Depression, Diabetes Distress and Medication Adherence Among Low-Income Patients with Poorly-Controlled Diabetes. J Gen Intern Med. 2015 Oct;30(10):1476–80. doi: 10.1007/s11606-015-3351-1 Epub 2015 Apr 28. ; PMCID: PMC4579205. 25917659 PMC4579205

[19] Mayer VL, McDonough K, Seligman H, Mitra N, Long JA. Food insecurity, coping strategies and glucose control in low-income patients with diabetes. Public Health Nutr. 2016;19(6):1103–1111. doi: 10.1017/S1368980015002323 26328922 PMC10270824

[20] Walker RJ, Grusnick J, Garacci E, Mendez C, Egede LE. Trends in Food Insecurity in the USA for Individuals with Prediabetes, Undiagnosed Diabetes, and Diagnosed Diabetes. J Gen Intern Med. 2019;34(1):33–35. doi: 10.1007/s11606-018-4651-z 30215178 PMC6318164

[21] Hernandez DC, Daundasekara SS, Arlinghaus KR, et al. Cumulative Risk Factors Associated with Food Insecurity among Adults who Experience Homelessness. *Health Behav Res*. 2019;2(1). doi: 10.4148/2572-1836.1033 31342011 PMC6656397

[22] Tong M, Tieu L, Lee CT, Ponath C, Guzman D, Kushel M. Factors associated with food insecurity among older homeless adults: results from the HOPE HOME study. *J Public Health (Oxf)*. 2019;41(2):240–249. doi: 10.1093/pubmed/fdy063 29617886 PMC6636692

[23] Bowen EA, Lahey J, Rhoades H, Henwood BF. Food Insecurity Among Formerly Homeless Individuals Living in Permanent Supportive Housing. Am J Public Health. 2019;109(4):614–617. doi: 10.2105/AJPH.2018.304927 30789774 PMC6417590

[24] Mullin B, Cervantes BS, Billimek J. Material Need Insecurity and Its Concurrent Barriers to Diabetes Management Among Low-Income Latino Adults Receiving Medical Care. *Diabetes Care* 1 March 2019; 42 (3): e31–e33. doi: 10.2337/dc18-1583 30674549 PMC6463549

[25] Chambers EC, McAuliff KE, Heller CG, Fiori K, Hollingsworth N. Toward Understanding Social Needs Among Primary Care Patients With Uncontrolled Diabetes. *Journal of Primary Care & Community Health*. January 2021. doi: 10.1177/2150132720985044 33467953 PMC7960895

[26] Gibson DM. Food Insecurity, Eye Care Receipt, and Diabetic Retinopathy Among US Adults with Diabetes: Implications for Primary Care. J Gen Intern Med. 2019 Sep;34(9):1700–1702. doi: 10.1007/s11606-019-04992-x ; PMCID: PMC6712119. 30972552 PMC6712119

[27] Stahre M, VanEenwyk J, Siegel P, Njai R. Housing Insecurity and the Association With Health Outcomes and Unhealthy Behaviors, Washington State, 2011. Prev Chronic Dis. 2015;12:E109. doi: 10.5888/pcd12.140511 26160295 PMC4509099

[28] Keene DE, Guo M, Murillo S. “That wasn’t really a place to worry about diabetes”: Housing access and diabetes self-management among low-income adults. Soc Sci Med. 2018;197:71–77. doi: 10.1016/j.socscimed.2017.11.051 29222997 PMC5771430

[29] CDC. Behavioral Risk Factor Surveillance System. BRFSS 2014 survey data and documentation. https://www.cdc.gov/brfss/annual_data/annual_2014.html

[30] CDC. Behavioral Risk Factor Surveillance System. BRFSS 2017 survey data and documentation. https://www.cdc.gov/brfss/annual_data/annual_2017.html

[31] Center for Disease Control and Prevention. Behavioral Risk Factor Surveillance System Operational and User’s Guide. Atlanta, GA: U.S. Department of Health and Human Services, Centers for Disease Control and Prevention, 2014.

[32] Njai R, Siegel P, Yin S, Liao Y. Prevalence of Perceived Food and Housing Security—15 States, 2013. MMWR Morb Mortal Wkly Rep 2017;66:12–15. doi: 10.15585/mmwr.mm6601a2 28081062 PMC5687269

[33] Centers for Disease Control and Prevention. Atlanta, GA: Centers for Disease Control and Prevention, US Department of Health and Human Services. Living with Diabetes: Education and Support. 2019. Available from: https://www.cdc.gov/diabetes/managing/education.html

[34] Campbell JA, Yan A, Egede LE. Community-based participatory research interventions to improve diabetes outcomes: a systematic review. Diabetes Educ. 2020 doi: 10.1177/0145721720962969 33353510 PMC7901040

[35] Marshall Sally M. “Intensive diabetes management for high-risk patients: how best to deliver?.” Diabetes care vol. 32,6 (2009): 1132–3. doi: 10.2337/dc09-0540 19460918 PMC2681023

[36] Cheyne K, Smith M, Felter EM, Orozco M, Steiner EA, Park Y, et al. Food Bank–Based Diabetes Prevention Intervention to Address Food Security, Dietary Intake, and Physical Activity in a Food-Insecure Cohort at High Risk for Diabetes. Prev Chronic Dis 2020;17:190210. 10.5888/pcd17.190210externalicon PMC6977780 31922370

[37] Sandel M, Hansen M, Kahn R, Lawton E, Paul E, Parker V, et al. Medical-legal partnerships: transforming primary care by addressing the legal needs of vulnerable populations. Health Aff (Millwood). 2010 Sep;29(9):1697–705. doi: 10.1377/hlthaff.2010.0038 . 20820029

[38] Ludwig J, Sanbonmatsu L, Gennetian, Adam E, Duncan GJ, Katz LF, et al. Neighborhoods, obesity, and diabetes—a randomized social experiment. N Engl J Med. 2011;365(16):1509–19. doi: 10.1056/NEJMsa1103216 22010917 PMC3410541

[39] Lim S, Miller-Archie SA, Singh TP, Wu WY, Walters SC, Gould LH. Supportive Housing and Its Relationship With Diabetes Diagnosis and Management Among Homeless Persons in New York City. Am J Epidemiol. 2019;188(6):1120–1129. doi: 10.1093/aje/kwz057 30834432

[40] Brown MM, Jason LA, Malone DK, Srebnik D, Sylla L. Housing First as an effective model for community stabilization among vulnerable individuals with chronic and nonchronic homelessness histories. Journal of Community Psychology. 2016.44(3), 384–390.

[41] Somers JM, Moniruzzaman A, Patterson M, Currie L, Rezansoff SN, Palepu A, et al. A Randomized Trial Examining Housing First in Congregate and Scattered Site Formats. PLoS One. 2017 Jan 11;12(1):e0168745. doi: 10.1371/journal.pone.0168745 ; PMCID: PMC5226665. United States Interagency Council on Homelessness. Housing First checklist: Assessing projects and systems for a Housing First orientation.2016 Sept 28. Retrieved from https://www.usich.gov/tools-for-action/housing-first-checklist. 28076358 PMC5226665

[42] Egede LE, Walker RJ, Linde S, Campbell JA, Dawson AZ, Williams JS, et al. Nonmedical Interventions For Type 2 Diabetes: Evidence, Actionable Strategies, And Policy Opportunities. Health Aff (Millwood). 2022 Jul;41(7):963–970. doi: 10.1377/hlthaff.2022.00236 35759702 PMC9563395

[43] Andrea LC, Schneider JS, Pankow GH, Selvin E. Validity and Reliability of Self-reported Diabetes in the Atherosclerosis Risk in Communities Study, American Journal of Epidemiology, Volume 176, Issue 8, 15 October 2012, Pages 738–743. 10.1093/aje/kws156 PMC3571247 23013620

[44] Fowles JB, Rosheim K, Fowler EJ, Craft C, Arrichiello L. The Validity of Self-reported Diabetes Quality of Care Measures. International journal for quality in health care: journal of the International Society for Quality in Health Care / ISQua. 1999. 11. 407–12. doi: 10.1093/intqhc/11.5.407 10561032

[45] Pierannunzi C., Hu S.S. & Balluz L. A systematic review of publications assessing reliability and validity of the Behavioral Risk Factor Surveillance System (BRFSS), 2004–2011. *BMC Med Res Methodol*. 2013. doi: 10.1186/1471-2288-13-49 23522349 PMC3622569

